# History of materials used for recording static and dynamic occlusal contact marks: a literature review

**DOI:** 10.4317/jced.50680

**Published:** 2013-02-01

**Authors:** Ashu Sharma, G R. Rahul, Soorya T. Poduval, Karunakar Shetty, Bhawna Gupta, Varun Rajora

**Affiliations:** 1 BDS, (MDS). Department of Prosthodontics, Bangalore Institute of Dental Sciences and Research Center, 5/3 Hosur Main Road, Opposite Lakkasandra Bus Stop. Wilson Garden, Bangalore, India; 2 BDS, (MDS). Professor and Head of Department of Prosthodontics. Bangalore Institute of Dental Sciences and Research Center, 5/3 Hosur Main Road, Opposite Lakkasandra Bus Stop. Wilson Garden, Bangalore, India; 3 BDS, (MDS). Professor. Department of Prosthodontics. Bangalore Institute of Dental Sciences and Research Center, 5/3 Hosur Main Road, Opposite Lakkasandra Bus Stop. Wilson Garden, Bangalore, India; 4 BDS. House Surgeon. Bangalore Institute of Dental Sciences. Bangalore, India; 5 BDS, MBA, (Ms HCA). Department of public affairs and administration. Cal. State. University east bay California, USA

## Abstract

In the discipline of prosthetic dentistry it is important not only to examine the occlusion, but to be able to record, store, and transfer the information. Over the years many occlusion testing materials have been used. It has been suggested the clinical recording and transfer of information using waxes and other occlusion recording materials have disadvantages relating to inaccuracy and problems of manipulation. Therefore, there has been introduction of many new systems for recording occlusion contacts to overcome such problems. The correct physiological recovery of occlusion posses as much a challenge as ever for every dentist and technician. Even the smallest high spots measuring just a few microns can cause dysfunctions like temporo-mandibular pain. Occlusal proportions are being constantly changed with every procedure. Therefore, an understanding of the synergy of the teeth in static and dynamic occlusion forms the basis of good dentistry. The purpose of this review article is to give and overview of the various materials and methods that have been used to record occlusal contact marks.

** Key words:**Occlusal contact marks, Occlusion indicators, Occlusion test materials, Occlusion recording materials.

## Introduction

Occlusal indicators are used to locate and define occlusal contacts (1). The occlusal contacts are exposed to constant change. Every tooth restoration, extraction and prosthetic care always implies a change in occlusal proportions. Traditional concepts of traumatic occlusal interferences involve a single anterior or posterior tooth, which is in “supracontact” during maximum intercuspidation or on excursive jaw movement. These two situations are collectively called occlusal interferences (2). An occlusal interference of only few microns can trigger severe irritation. In order to avoid any unpleasant sensation, the patient will probably not bite on the new dental bridge but rather move his lower jaw into a physiologically unsound position. The new bite of convenience causes irregular muscle activity which can eventually lead to temporomandibular joint pain and myalgia.

Over the years various materials and methods have been used to detect high spots. Achieving occlusal markings over some restorations such as gold, metal alloys and ceramics and on moist occlusal surfaces has been a real challenge. For an accurate examination of occlusion in prosthodontic treatment, it is important to understand the patterns of tooth contact, properties of materials and methods used to record these tooth contacts (3).

## Classification of tooth-contact patterns

The tooth contact patterns were classified into four groups as follows:

1. Cuspid protected occlusion: contact of canines on the working side

2. Group function occlusion: contact of canines, premolars, and/or molars, or contacts of premolars and molars on the working side only

3. Full balanced occlusion: tooth contact patterns with group function or cuspid protected occlusion on the working side plus multiple tooth contacts of posterior teeth on the nonworking side

4. Others: occlusal patterns other than those described. Contact of incisor teeth, if any, were included in this classification (3).

Hellman described four ways in which teeth contact:

1. surface

2. cusp tip and fossa

3. ridge and groove

4. ridge and embrasure.

He stated that there are 138 possible contacts in the dentition with normal occlusion. Experimentally, he found that 90% of the total units actually make exact contact in dentitions with normal occlusion.

The sensitivity and reliability of the techniques currently used for occlusal analysis depend on the thickness, strength, and elasticity of the recording materials, as well as the oral environment and clinician’s interpretation (1,4-5). Both qualitative and quantitative methods are used for the evaluation of occlusal relationships (5).

With the qualitative method, only the localization of the occlusal contact points can be determined; the sequence or density of the contacts cannot be evaluated. Although an opinion can be derived from the density of the contacts according to the darkness of the marks, this is not a precise criterion for evaluation. Wax, articulating paper, foils, and even silk strips are used for qualitative analysis (1). With the quantitative method of evaluating occlusal relationships, the sequence and density of the contacts can be differentiated. Photo-occlusion and the T-Scan system (Tekscan Inc., Boston, Mass.) are quantitative measures for determining occlusal relationships (1,6-12).

## Occlusion indicating materials and techniques used in the past and present

There are various materials that have been used in the past and present to detect the occlusal contact points. These have been listed below irrespective of the year of their use and their accuracy, sensitivity and reproduci-bility ability ( Table 1).

**Table 1:**
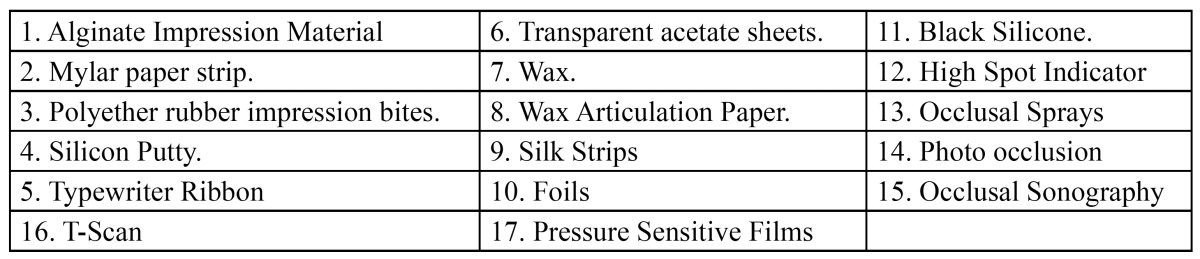
Various occlusal recording materials.

## Alginate Impression Material Index

Korioth (13) reported on the number and location of occlusal contacts in intercuspal position using alginate impression material. A technique suggested and used by Ingervall, (14) using indexes of alginate (irreversible hydrocolloid) impression material was applied to record the number and location of posterior occlusal tooth contacts, including canines. The selected subjects rested their backs and heads on a reclined dental chair (approximately 30 degrees to the floor). After spatulation, the impression material was applied to the occlusal surfaces of all lower canines, premolars, and molars on both sides. Subjects were then instructed to close the mouth slowly and press the teeth together with light to moderate pressure until the impression material was set. All impressions were made at the same period of time during the day. After their careful removal, the left and right indexes were examined against light, and the number and location of perforations were registered as occlusal tooth contacts for each subject. The chi-square test was used to determine any statistically significant differences between the right and left sides for the observed number and location of contacts.

## Mylar Paper / Shimstock films

The shim stock 8 mm in width and was positioned over the tooth evaluated. When the participants close in Intercuspal Position, teeth holding the shimstock were considered to have occlusal contact with their antagonists. In this manner, proceeding tooth by tooth around the dental arch, the dentist identifies the teeth with contact (15-16).

Metallic shimstock-films (Bausch Arti-Fol, Bausch articulating paper Inc, Nashua, NH, USA) is made of metallic polyester-film of 12microns thick. A combination of color coating and metallic film offers extra advantage of high spot precision over conventional shimstock films.

Anderson et al. (15) reported on the reliability of dentists’ ability to evaluate occlusal contacts in the intercuspal position. They compared an articulating paper method against a Mylar paper method and found the latter to be more reliable. In a study of the thickness, strength and plastic deformation of occlusal registration strips used to detect occlusal contacts patterns, Halpern et al. (17) found that some recording methods (those with a stiff marking media) induced artifacts in the contact detection process. These investigations are good beginning if a better understanding of the weakness and strength of the entire clinical occlusal examination are over to be achieved.

## Polyether Silicon Impression Bites

Durbin and Sadowsky (18) described a silicone impression material method for examining occlusal contact patterns before and after orthodontic treatment. Although this method has good accuracy, it is impractical. However, it might serve as a gold standard against which other, easier- to-use clinical methods could be tested (2). Polyether rubber impression bites were used to record occlusal contacts. The locations of the contacts were then transferred to study models.

## Silicon Putty Material

In an occlusal study Ziebert and Donegan (19) used silicone putty to check occlusal contacts after occlusion adjustments in their patients. Silicone putty interocclusal records were made in the intercuspal and in the retruded contact position immediately after each set of impressions were made. Each record was repeated until two identical records of each position were obtained. The record for the intercuspal position was made with no guidance provided by the examiner, whereas firm mandibular guidance was provided for the retruded contact position records. The interocclusal records were trimmed and placed on the casts. The location of tooth contacts was observed as perforations in the silicone putty records. Liqua-Mark, a color indicator, was painted into the perforations of each record with a fine camelhair brush to produce discrete markings on the casts. Different colors of the color indicator were used to differentiate between the intercuspal and retruded contact positions.

It is not clear how the decision that the records were identical was verified, nor was intraoperator reliability tested (20). Millstein (21) used photographic method to compare occlusal contact marks made on acrylic resin casts with perforations that occurred in a silicone interocclusal record. The author concluded that the occlusal contact markings might be specific for, and a product of, the occlusal indicator papers and might not represent the contact surfaces determined by the silicone interocclusal records.

## Typewriter Ribbon

Ziebert and Donegan (19) used typewriter ribbon to mark supracontacts or occlusal interferences in their patients for occlusal adjustments. Interferences were marked with typewriter ribbon and contacts verified with 0.00l-inch shim stock. The adjustment procedure basically that of Schuyler (22-23), following the M. U.D.L. rule for the retruded position, the B.U.L.L. rule for the retruded position , the B.U.L.L rule for the working movement, and the D.U.M.L. rule for protrusion. Nonworking interferences were eliminated so as to maintain at least one centric stop on each tooth.

## Transparent Acetate Sheet

Davies et al. (24) described a clinical method termed the occlusal sketch technique as a means of recording occlusal contacts. The sketch consists of an acetate sheet on which a schematic representation of the teeth is drawn, including the occlusal surfaces of the posterior teeth, the palatal surfaces of the maxillary anterior teeth and the labial surfaces of the mandibular anterior teeth. The same authors concluded that this technique demonstrated interoperator and intraoperator reliability in recording occlusal contacts in vitro. The aim of the occlusal sketch technique is to provide a simple and reliable means of recording and transferring information about the location of marked occlusal contacts. It may also be used by the technicians to verify occlusal contacts when articulating casts and fabricating indirect restorations (24).

## Wax

Ehrlich and Taicher (25) recorded occlusal contacts by placing the wax on the occlusal surfaces of the maxillary posterior teeth and having the patient close into maximum intercuspation. The wax occlusal records were subsequently examined in front of a light screen. After recording the quality of occlusal contacts, each registration was placed on the diagnostic cast to visualize and verify the exact location of each contact (supracontact, contact, and near contact).

However, this methodology was not tested for either interoperator or intraoperator reliability (20). Murray (26) suggested that the clinical recording and transfer of information using waxes have disadvantages relating to inaccuracy and problems of manipulation.

## Wax Articulation Paper

Articulating papers frequently are used to detect high spots, the width, thickness and dye type of the articulating paper enables it to leave a mark of either a point or a surface (1). The color coating of many articulating papers consists of waxes, oils and pigments, a hydrophobic mixture which repels saliva (hydrophilic) consisting mainly of water. High spots can be detected easily as dark marks and contacts as light marks. Articulating paper come sin strips and horse shoe shaped sheets (Bausch articulating paper Inc, Nashua, NH, USA). When grinding selectively it should be noted that only dark colored spots should be ground.

The major disadvantages of articulating papers have been that they can be easily ruined by saliva, are thick, and they have a relatively inflexible base material; all of these factors result in a greater number of pseudo contact markings (27-28).

Few manufacturers have produced articulating films with an additional emulsifier (Bausch articulating paper Inc, Nashua, NH, USA) which gives these films certain bonding properties on moist occlusal surfaces. They have added special bonding agent- transculase (Bausch articulating paper Inc, Nashua, NH, USA), or wetting agents like lecithin (27) to articulating paper coating. The first test is made with blue articulating paper (200microns). Spots are immediately evident. The bonding agent, transculase, is also transferred as a fine coating. The next step is to take a thin film (preferably red, 8microns) because of its intensity and excellent contrast with blue . The color transfer of these film are considerably improved with the help of transculase coating.

## Silk Strips

Some researchers have stated that silk strips are the best material for indicating occlusal contacts. Articulating silk is made from high quality natural silk (Bausch articulating silk, 80 microns, Bausch articulating paper Inc, Nashua, NH, USA). Natural silk consists of so-called fibrils, a tube-shaped protein structure which, because of its composition, has an extremely high color reservoir capacity. This silk is highly tear-resistant and, because of its low thickness and good flexibility, adapts perfectly to cusps and fossae. The marking of silk is extremely precise. Because of their texture, soft indicator materials do not produce pseudo contact markings.

However, silk strips can lose their marking abilities when their stain components are dried, and they also can be ruined by saliva. It is therefore advisable to store them in a cool, closed environment (1,28).

## Foil

Foils are the thinnest indicator materials They give more accurate readings than paper and silk. However, under reduced pressure and on glossy surfaces, their marking capacity is less evident. This means that greater pressure must be applied for the clinical use of foils (28).

## Black Silicone

Occlusal registration to record tooth contacts with black silicone was carried by Takai et al. in 1993. A black silicone impression material (G. C. Dental Industrial Corp.) was mixed according to the manufacturer’s specifications and applied with a syringe on all occlusal surfaces of the teeth. The patients were then asked to close their mouths slowly in the intercuspal position, immediately slide the mandible to the designated lateral position 1 or 2, and maintain that position until the black silicone impression material had set. The occlusal registration was carefully removed and observed against the light to verify the tooth contact sites. Perforations or translucent areas were identified as tooth contacts (3).

## High Spot Indicator

It is a liquid contact color (Arti-spot, Bausch articulating paper Inc, Nashua, NH, USA) which is applied to the test surface with a brush . The solvent evaporates in seconds, leaving a thin film (3 microns thick). Every contact destroys skin color exactly at the point of contact. The base material then shines through and high spots can easily be detected. It can also be used to test for high spots on highly polished occlusal surfaces such as gold or ceramic. The food dye contained in the solvent is completely safe. The layer can easily be removed after use with hot water or alcohol.

## Occlusal Sprays

These are universal color indicator to test occlusal contacts. They are easy to administer (Arti-Spray, Bausch articulating paper Inc, Nashua, NH, USA) and leaves a thin colored film which can easily be removed with water, leaving no trace of residues. They are applied at a distance of 3-5 cm onto the occlusal surface. When testing occlusion all contact points will be immediately visible. These are available in colors: red, blue, green and white.

## Photo-Occlusion

In a photo occlusion system, a thin photoplastic film layer is placed on the occlusal surface of the teeth; the patient then is asked to occlude on the film layer for 10 to 20 seconds. The film layer is removed from the mouth and inspected under a polariscope light. This technique is reported to be “difficult to apply”. Gazit et al. compared the results of occlusal examination of 11 dental students by means of photoelastic wafer and articulating paper. The results were transferred to a graphic occlusal scheme. Neither technique was found to be highly reproducible (29).

## Occlusion Sonography

The first studies to detect tooth contact by the sounds generated during mouth closure began to appear in the literature in the 1960s (30) one commercial device was produced in the mid 1980s called “Dental Sound Checker” (Yoshida Dental Trade Distributing Co Ltd, Tokyo, Japan). The device, based on the principles put forth by Watt, was developed to evaluate occlusal contact sound paterns during closure in an attempt to detect occlusal disturbances. Klifune et al (31) measured the duration of the occlusal sound in a single subject before and after occlusal adjustment and reported a clear decrease in the duration of the occlusal sound with adjustment.

## T-Scan

The development of a prototype computerized occlusal analysis (T-Scan; Tekscan Inc, South Boston, Mass) was reported by Mannes et al. (6) The T-Scan instrument was designed to examine and record occlusal contacts by computer analysis of information from a pressure-sensitive film. The T-Scan system is purported by the manufacturer to digitally record both the location and timing of tooth contacts. The tooth contact information is presented by demonstrating moments of time in the sagittal axis and transverse axis of the occlusal plane. Time moments are defined as the sum of distances of the tooth contacts in millimeters from the x or z axis of the occlusal plane multiplied by their relative time value (1-sec) and divided by the sum of the onset times. The manufacturer purports that, when the time moments in these axes are analyzed, an occlusion can be uniquely described (20).

In this system, electrical resistance develops with the applied force. When the patient occludes on the sensor, the particles come together in the force applied areas, diminishing the electrical resistance. The u-shaped sensor foil is 60microns thick, consists of an X-Y coordinate system with 1500 sensitive receptor points made of conductive ink, and is subject to elastic deformation (9-12). When an operator properly uses this technology, mark size, mark color-depth, donut-shaped halo contacts, as well as other color and mark appearance characteristics, are ignored as force indicators and used only as contact locators (32).

The first occlusal contact that results when the mandible is closed on a correct centric relation axis is known as the centric relation prematurity. This procedure (T-Scan) combines bimanual manipulation with the simultane-ous recording of the sequence of resultant tooth contacts using a computerized occlusal analysis system. (33).

Several researchers have reported that the sensors do not have the same accuracy among themselves and have fewer contacts than conventional methods, such as articulating papers (11,34). However, it has been shown that the pressure- sensitive film method is not as accurate as the silk ribbon and detecting occlusal contacts (35). For this reason, it appears that the clinical applicability of the T-Scan system is limited. The sensitivity of the T-scan sensors has been reported to decrease or disappear when the sensors are used more than once (11).

Mizui et al. (36) measured the timing and force of occlusal contacts in both 60 normal subjects and 5 patients with an unspecified craniomandibular disorders (CMD) using the T-Scan system. They reported that in the normal subjects the timing and force of occlusal contacts were symmetrical and the center of effort was located in the first molar region. For patients with CMD, the timing and force of occlusal contacts were asymmetric and the center of effort was not always located in the first molar region, as determined with the T-scan system.

## Pressure Sensitive Films:

A newer but essentially similar device has been introduced (Dental Prescale, Fuji Film, Tokyo, Japan). This device also records the location and force of contacts with the force sensitive film. Hattori et al. (2,37) evaluated the reliability of this device for occlusal force measurement both on a subject and on casts. They reported the linear relationship between the applied and measured loads.

Araki et al. (2) used this device in 5 patients with TMD and evaluated the distribution and area of the tooth contacts and the total occlusal force. The primary limitation of the contact sensor and the pressure sensitive film device is that the recording medium is far too thick and results in heavier contacts on the posterior teeth than the anterior teeth. Further, this sensor thickness disturbs the persons finding attempts to close into the intercuspal position. This is because a study on interocclusal thickness discrimination has shown that aluminium foil as thin a 20 micrometer can give bite-disturbing proprioceptive information to a subject (38).

## Conclusion

When used more than once, the articulating papers, foils, silk strips, and T-Scan system tested as occlusal indicators were associated with different rates of decrease in contact numbers. The success of the T-Scan system was negatively affected by repeated use of the sensors. Occlusal records obtained in wet and dry environments were significantly different. Occlusal contact numbers increased greatly when the teeth were dry; drying the mouth thoroughly before testing therefore may affect the success of occlusal analysis. Every material has its own limitations in someway, be it age-old materials like wax or impression materials or the latest technology of T-Scan. The choice to use any one out of the above mentioned materials depends upon the clinical situation, clinician’s choice and expertise, economics and comfort.
